# N-glycosylation in the SERPIN domain of the C1-esterase inhibitor in hereditary angioedema

**DOI:** 10.1172/jci.insight.185548

**Published:** 2025-01-16

**Authors:** Zhen Ren, John Bao, Shuangxia Zhao, Nicola Pozzi, H. James Wedner, John P. Atkinson

**Affiliations:** 1 Department of Medicine, Division of Allergy and Immunology, Washington University School of Medicine, St. Louis, Missouri, USA.; 2 Department of Molecular Diagnostics and Endocrinology, The Core Laboratory in Medical Center of Clinical Research, Shanghai Ninth People’s Hospital, Shanghai Jiaotong University School of Medicine, Shanghai, China.; 3 Department of Biochemistry and Molecular Biology, Edward A. Doisy Research Center, Saint Louis University School of Medicine, St. Louis, Missouri, USA.; 4 Department of Medicine, Division of Rheumatology, Washington University School of Medicine, St. Louis, Missouri, USA.

**Keywords:** Immunology, Genetic variation, Glycobiology, Serpins

## Abstract

Hereditary angioedema is an autosomal dominant disorder caused by defects in C1-esterase inhibitor (C1-INH), resulting in poorly controlled activation of the kallikrein-kinin system and bradykinin overproduction. C1-INH is a heavily glycosylated protein in the serine protease inhibitor (SERPIN) family, yet the role of these glycosylation sites remains unclear. To elucidate the functional impact of N-glycosylation in the SERPIN domain of C1-INH, we engineered 4 sets consisting of 26 variants at or near the N-linked sequon (NXS/T). Among these, 6 are reported in patients with hereditary angioedema and 5 are known C1-INH variants without accessible clinical histories. We systematically evaluated their expression, structure, and functional activity with C1s̄, FXIIa, and kallikrein. Our findings showed that of the 11 reported variants, 7 were deleterious. Deleting N at the 3 naturally occurring N-linked sequons (N238, N253, and N352) resulted in pathologic consequences. Altering these sites by substituting N with A disrupted N-linked sugar attachment, but preserved protein expression and function. Furthermore, an additional N-linked sugar generated at N272 impaired C1-INH function. These findings highlight the importance of N-linked sequons in modulating the expression and function of C1-INH. Insights gained from identifying the pathological consequences of N-glycan variants should assist in defining more tailored therapy.

## Abstract 2

N-glycosylation in the SERPIN domain of C1-INH is critical in assisting protein folding and function. Altering/deleting N-linked sugar at the glycosylation sites leads to pathologic consequences.

## Introduction

Hereditary angioedema (HAE) is an autosomal dominant (AD) disorder caused by defects in the *SERPING1* gene encoding C1-esterase inhibitor (C1-INH) (1). C1-INH is a multifunctional protein that inhibits the contact system, fibrinolytic pathway, and complement activation. A dysfunctional C1-INH results in poorly controlled activation of the kallikrein-kinin (PKa) system, leading to bradykinin overproduction, which is known to be the primary mediator of recurrent and unpredictable soft tissue swelling. Laryngeal edema is associated with the risk of asphyxiation, which can be life threatening (1).

HAE is categorized based on a low laboratory plasma C1-INH antigen level (TYI) or normal antigen but with a dysfunctional C1-INH protein (TYII) (2). This AD disorder results in haploinsufficiency that would be expected to produce 50% of the normal level of C1-INH in plasma (3). However, most patients with HAE present with plasma C1-INH antigen levels of approximately 5% to 30% of normal (4). The cause of these disproportionally decreased C1-INH plasma levels remains largely unknown (5, 6). Furthermore, the severity of clinical symptoms does not necessarily correlate with the antigenic levels of C1-INH (1).

Over 1,100 variants in the *SERPING1* gene have been reported and more than one-half are missense single nucleotide variants (SNVs) (7). Interpreting the pathogenicity of these *SERPING1* variants can be difficult. This is in part due to the limitations of in silico prediction tools and of the genetic variant databases. Relative to the latter, each genetic variant database provides its own unique annotations and datasets, presenting a challenge when integrating clinical and genetic data from different sources.

**Figure 1:**
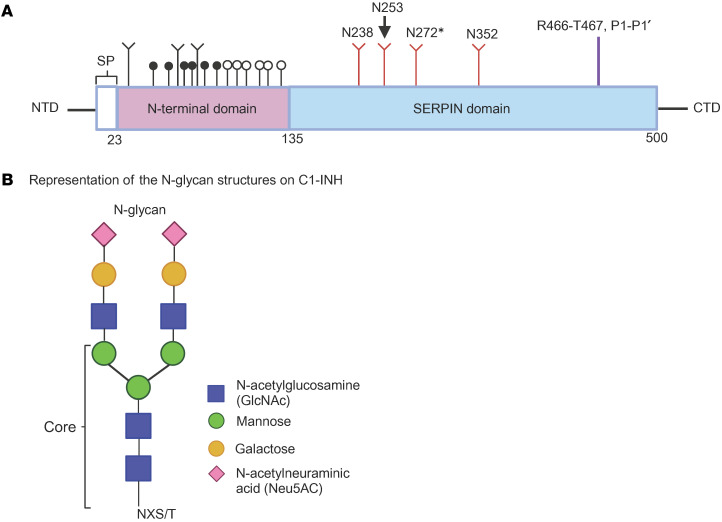
Schematic representation of protein domains and N-glycan for C1-INH. (**A**) The N-terminal domain includes resides 23–134 (pink). The C-terminal SERPIN domain consists of residues 135–500 (blue). Signal peptide (SP) contains 22 residues and is included in the numbering. Branched symbols, N-glycosylation (*n* = 7); black circles, verified O-glycosylation sites (*n* = 7); white circles, potential O-glycosylation sites (*n* = 7). N-glycosylation sites in the SERPIN domain are highlighted in red. Protease cleavage site, P1-P1′ highlighted in purple. NTD, amino-terminal domain; CTD, carboxyl-terminal domain. (Adapted from *The Complement Factsbook*, Figure 23.1). (**B**) Representation of the N-glycan structure on C1-INH. Based on a mass spectrometry study, the majority of the N-glycans on N238, N253, and N352 are biantennary, approximately 80%, with HexNAc_4_Hex_5_NeuAc_2_ being the most abundant N-glycan form (14). *The N272 glycosylation site has not been confirmed by mass spectrometry.

**Figure 2:**
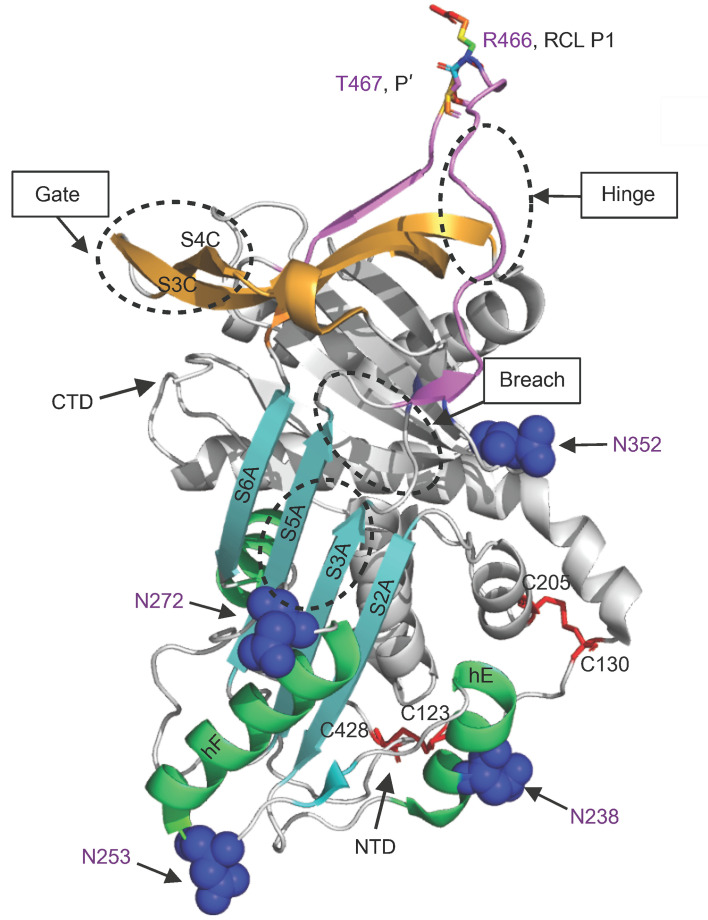
Illustration of key domains in C1-INH. The reactive center loop (RCL) is in pink. The regions involved in SERPIN function are labeled. The P15-P9 portion of the RCL, the hinge domain, is highly conserved and facilitates the insertion of RCL into β-sheet A (SA). The breach region lies on the top of SA, the initial insertion site of RCL. The shutter domain, composed of S3A and S5A, is in the center of SA and facilitates the RCL insertion. The gate region consists of strands 3 and 4 from β-sheet C (S3C and S4C). N-glycosylation sites are shown as blue spheres. The P1 and P′ are displayed as rainbow sticks and are responsible for trapping the target protease. Strands of central SA are in cyan. The 2 disulfide bridges are labeled and colored in red. Protein structures used for modeling were obtained from the PDB database (PDB: 5DU3). The figure was generated using Pymol (3.0) and serves as a model for structure analyses in Figures 4–7. NTD, amino-terminal domain; CTD, carboxyl-terminal domain.

**Figure 3:**
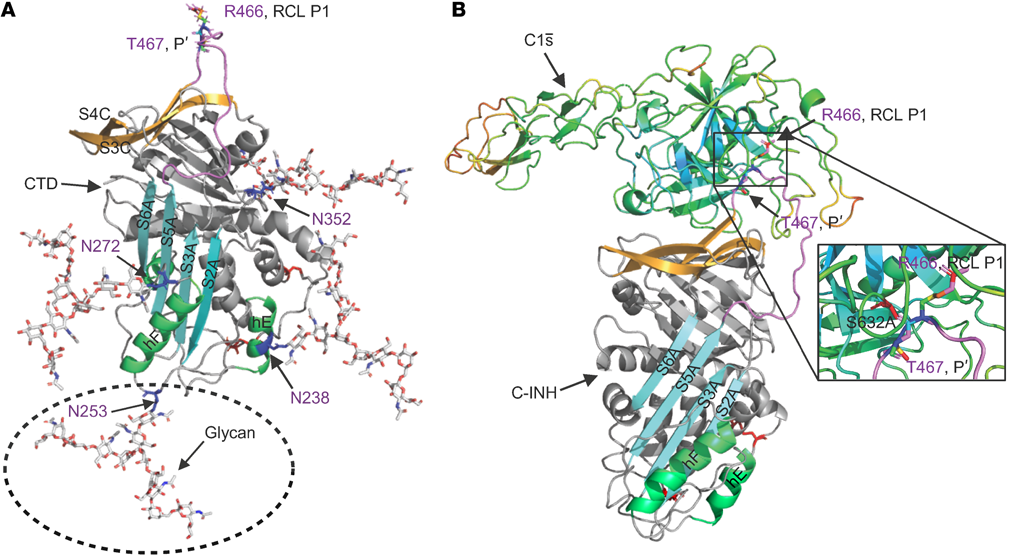
Illustrations of N-glycans on the SERPIN domain in C1-INH and a C1s̄/C1-INH complex. (**A**) Molecular model of N-glycans on the SERPIN domain in C1-INH (PDB: 5DU3). Molecular modeling was performed on the GlyCAM-Web tool, Glycoprotein Builder (https://glycam.org). The N-linked glycan structure was generated based on mass spectrometry results (14). The N-GlcNAc linkage conformation was based on the simulation generated from Glycoprotein Builder. (**B**) Structure of a C1s̄/C1-INH complex (PDB: 8W18). The structure of active C1s̄ is shown in a cartoon representation in rainbow. The RCL is in violet. The P1 R466 and P1′ T467 residues are displayed as rainbow sticks and are responsible for trapping C1s̄. The disulfide bridges are labeled and colored in red. Recombinant C1s̄ harbors an S632A mutation, making it catalytically inert (PDB: 8W18) (9). Figures were produced using PyMOL (3.0).

C1-INH protein contains 500 aa that includes a 22 aa signal peptide, an amino-terminal domain (NTD) consisting of 112 aa, and a carboxyl-terminal domain (CTD), also named the serine protease inhibitor (SERPIN) domain, containing 366 aa (Figure 1). The SERPIN domain is highly conserved, comprising 9 α-helices, 3 β-sheets, and a reactive center loop (RCL) (Figure 2) (8). The target protease binds to the RCL, forming a reversible “Michaelis complex,” and then cleaves the P1-P1′ scissile bond (R466-T467) (9). Upon cleavage, the SERPIN-protease complex forms a covalent bond between the P1 residue of the RCL and the active-site serine of the protease (Figures 2 and 3) (9, 10). The cleaved RCL undergoes drastic conformational changes and inserts into β-sheet A (SA), becoming an additional strand, S4A, in the SA domain. This process leads to the formation of an irreversible C1-INH–protease complex (9). In contrast with the well-defined structure and function of the SERPIN domain, the NTD is poorly understood. Crystal structure models of C1-INH are only available for the CTD, as the first 100 aa from the NTD were not included in the studied structures (Figures 2 and 3) (11).

In typical glycoproteins, the glycans can constitute up to approximately 20% of the total weight (12). However, C1-INH is a heavily glycosylated protein, with an estimated relative electrophoretic mobility (M_r_) of 105,000, in which approximately 50% of the mass results from glycosylation (13). C1-INH contains 6 N-glycosylation sites, 3 of which are in the NTD, while the others are in the SERPIN domain (Figure 1 and Figure 3A). The NTD also contains up to 26 O-glycosylation sites (Figure 1) (14). Even though most glycans reside in the NTD, the recombinant C1-INH without an NTD appears to have preserved SERPIN function, suggesting that N-glycosylation on the SERPIN domain may play a crucial role in stabilizing the C1-INH protein, thereby assisting in protein folding and function (10, 15, 16).

N-linked glycosylation is the predominant type of glycosylation in eukaryotic cells (17). This process is catalyzed by an oligosaccharyltransferase (OST) through recognition of an N residue in the canonical NXS/T sequon that promotes attachment of a preassembled oligosaccharide to N using an N-glycosidic bond (18, 19). The conserved NXS/T sequon requires that an X be at the +1 position, which can be any aa except for P, and either an S or T at the +2 position of the accepting sequon (18, 19).

This study investigates the impact of *SERPING1* variants at the N-glycosylation sites in the SERPIN domain. We hypothesized that deleting the N or S/T residue in the NXS/T sequon could destabilize the protein due to it lacking glycosylation modifications. Here, we present a systematic evaluation of 4 sets of 26 *SERPING1* variants at or near the N-glycosylation sites in the SERPIN domain. Among these, 6 variants are reported in HAE patients, while 5 variants are known human *SERPING1* variants lacking an accessible clinical history. These studies not only shed light on the role of N-glycosylation in the SERPIN domain of C1-INH, but also validate how patient variant modeling can help identify pathological consequences and potentially point to improved clinical therapeutic strategies.

## Results

This study investigates the impact of N-glycosylation on the SERPIN domain of WT C1-INH as well as the effects of known human variants through analysis of 4 sets of variants either at or near N-glycosylation sites (N238, N253, N352, and N272). Each set of variants includes an N to A substitution, an N deletion, and an S deletion at the +2 position of the sequon. In addition, we constructed 11 *SERPING1* variants in close proximity to the N-glycosylation sites based on the published literature, the Genome Aggregation Database v4.1 (gnomAD), and the Leiden Open Variation Database v3.0 (LOVD). Among these, 6 variants are reported in patients with HAE. The variants were expressed recombinantly in human embryonic kidney 293T (HEK293T) cells containing the intact glycosylation machinery. The protein expression levels of the C1-INH variants were compared to WT C1-INH. We then analyzed the impact of these variants on protein structure and interactions with its functional substrates, including activated C1s (C1s̄), FXIIa, and PKa (see Table 1) (20, 21).

### N-glycosylation site N238

#### Experimental design.

**Figure 4:**
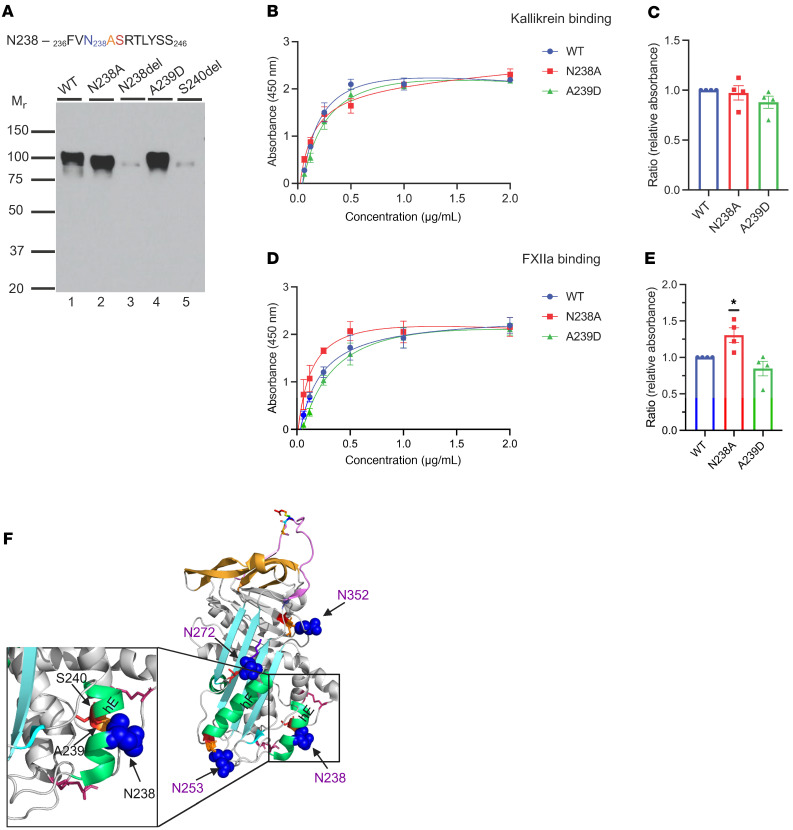
N238 glycosylation site variants. (**A**) Western blot (WB) analysis of supernatants from transfected WT C1-INH and variant constructs of N238 under reducing conditions. The recombinant expression of N238A and A239D was comparable to WT (see Table 1). The secretion of N238del was markedly decreased compared with that of WT. The consensus sequence of N238 glycosylation NXS/T is highlighted in blue, orange, and red. (**B**–**E**) Functional analysis of N238 variants. (**B** and **D**) Absorbance is plotted against protein concentration. (**C** and **E**) Relative absorbance (RA) was computed as the absorbance of the variant divided by the absorbance of the WT at concentrations of 1 μg/mL, 500 ng/mL, 250 ng/mL, and 125 ng/mL. Data represent 3 separate experiments, with bars corresponding to SEM. *P < 0.05 for the percentage difference in FXIIa binding between N238A and WT by 1-way ANOVA with Dunnett’s multiple-comparison test. The *P* value for the percentage difference in FXIIa binding between A239D and WT is 0.358. For PKa binding, the *P* values for the percentage differences of N238A and A239D compared with WT are 0.918 and 0.254, respectively. (**F**) Structural analysis of N238 (PDB: 5DU3). N238 is located on the surface of helix E (hE). N238del results in the disruption of the consensus sequence NXS, which is required for the attachment of N-glycan. In the absence of glycosylation, N238del likely leads to protein misfolding.

The N-glycosylation sequon at N238 employs a consensus sequence, _236_FVN_238_ASRTLYSS_246_ (Figure 4). To investigate the impact of impaired glycosylation at position N238 on C1-INH expression and function, we created N238A and N238del at the N-linked glycan site and S240del at the +2 position of the sequon. Additionally, we engineered A239D, which is reported in gnomAD.

#### Genetic analysis.

A239D was reported with an allele frequency (AF) of 0.0004% in the gnomAD and Mastermind databases. This variant has not been reported in other databases, including VarSome, LOVD, or ClinVar. In silico tools predicted this variant to be benign.

#### Structure.

N238 is located on the surface of helix E (hE), which connects strands 1 and 2 of β-sheet A (S1A and S2A) (Figure 2). The hE plays a crucial role in facilitating the conformational change during the insertion of the RCL between S3A and S5A. The deletion of N238 alters the hydrophilic surface of hE and thereby alters the packing of the hE, leading to protein misfolding (Figure 4F).

#### Antigenic and functional analyses.

We expressed N238A, N238del, A239D, and S240del separately in HEK293T cells. N238A demonstrated a normal protein secretory pattern, while secretion of the recombinantly produced N238del and S240del was barely detectable. The secretion level of A239D was approximately 30% higher than WT (Table 1 and Figure 4A). The binding affinity of N238A and A239D for C1s̄, FXIIa, and PKa was comparable to WT (Figure 4, B–E).

#### Implication.

The N-linked glycan site at N238 employs an NXS sequon. We hypothesize that deleting the N or S residue likely destabilizes the protein because it lacks proper posttranslational glycan structure modification. In the case of the N238 sequon, the deletion of N or S abolished protein expression. Replacing N238A led to normal recombinant protein expression and preserved its function (Figure 4).

A239D is at the +1 position (X) of the NXS sequon, so it can be any aa except P without affecting the recognition of glycan attachment. Compared with WT, A239D exhibited the same M_r_ and the secretory level of A239D was higher (Table 1 and Figure 4A). A239D demonstrated a normal (comparable to WT) binding affinity for FXIIa and PKa (Figure 4, B-E).

### N-glycosylation site N352

#### Experimental design.

**Figure 5:**
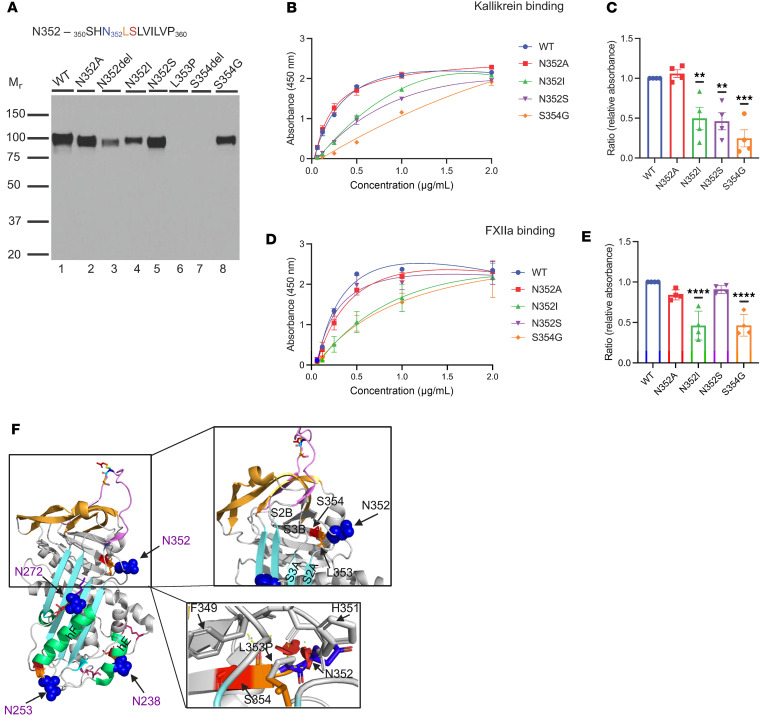
N352 glycosylation site variants. (**A**) WB analysis of supernatants from transfected WT C1-INH and variant constructs of N352 under reducing conditions. One representative experiment of 3 is shown. Recombinant expression of N352A, N352S, and S354G was comparable to WT, whereas N352del, L353P, and S354del were barely secreted. The secretion of N352I was decreased (see Table 1). The consensus sequence of N352 glycosylation (NXS/T) is highlighted in blue, orange, and red. (**B**–**E**) Binding analysis of N352 glycosylation site variants. (**B** and **D**) Representation of PKa and FXIIa binding of N352A, N352S, N352I, and S354G compared with WT. (**C** and **E**) N352I and S354G demonstrated impaired binding to both PKa and FXIIa. N352S showed decreased binding to PKa, but not to FXIIa. Data represent mean ± SEM of 3 independent experiments. **P < 0.01, ***P < 0.001, ****P < 0.0001 by 1-way ANOVA with Dunnett’s multiple-comparison test. (**F**) Structural analysis of N352. N352 is located in the loop connecting strands 2 and 3 in β-sheet B (S2B and S3B), which is the hydrophobic core of C1-INH. N352del disrupts the packing of the hydrophobic core and leads to protein misfolding. The substitution with L353P can cause structural disturbances by disrupting hydrogen bridges and affecting the packing of the loop between S2A and S3A, thus leading to protein misfolding. Yellow dashed line, hydrogen bond.

The conserved consensus sequence at the N352 position is _350_SHN_352_LSLVILVP_360_ (Figure 5A). We constructed N352A, N352del, and S354del at the sequon. Based on the data from gnomAD and LOVD, we also created 4 additional variants: N352I, N352S, L353P, and S354G.

#### Genetic analysis.

N352S and S354G are reported in gnomAD with an AF of 0.00061% and 0.00065%, respectively. In silico predictions for N352S suggest that this change is benign, whereas S354G has conflicting predictions. S345G has a REVEL score of 0.845 and a CADD score of 25.33, indicating a potentially deleterious effect. Other scores, such as pholyP, Pangolin, and SpliceAI, indicate that this change is benign. N352I and L353P are reported in an HAE cohort from Germany, but are not included in gnomAD (22, 23).

#### Antigenic and functional analyses.

Variants N352A, N352del, N352I, N352S, L353P, S354del, and S354G were individually expressed in HEK293T cells (Table 1). The secretion of recombinantly produced N352A, N352S, and S354G was comparable to WT. The secretion of N352del was markedly decreased, being approximately 10% compared with WT. The secretion of N352I was reduced to approximately 50% compared with WT (Table 1), and L353P and S354del were undetectable. N352I, N352S, and S354G showed a slightly lower M_r_ on Western blot (WB) compared with WT, approximately 100,000 and 105,000, respectively. Functional analyses revealed that N352A has a normal binding to C1s̄, PKa, and FXIIa. N352S demonstrated a normal binding to C1s̄ and FXIIa, but reduced binding to PKa. Both N352I and S354G showed mildly reduced binding of FXIIa and PKa, but not to C1s̄ (Table 1 and Figure 5, B–E).

#### Structure.

N352 is located at the end of loop in β-sheet 2B (S2B) as it transits into S3B (Figure 5F). It is situated in the center of the breach region, where the RCL inserts into the shutter domain. Deleting N352 destabilizes the packing of S2B and S3B, which may lead to protein misfolding. The replacement of N352 with I is unfavorable. Asparagine (N) is a polar aa and prefers to be on the protein surface. Isoleucine (I) is a hydrophobic aa, which is challenging to adapt to an α-helical confirmation and prefers to lie within β sheets. The substitution of N with I at N352 may change the orientation of the loop, resulting in protein misfolding and thereby leading to decreased protein expression (Figure 5A). In the case of N352S, both N and S are polar. Therefore, substituting N for S is structurally tolerable but changes the glycosylation of N352. S354 is located at the tip of S3B. Replacing a buried S354 with G would markedly reduce the side-chain volume. This could cause disruption and alter the packing of the S3B region, thereby leading to C1-INH dysfunction (Figure 5F).

#### Implication.

The N-linked glycan site at N352 employs an NLS sequon and the deletion of N or S in the sequon results in drastically decreased protein expression, which is similar to what we observed at the N_238_AS glycosylation site (Table 2). Additionally, replacing N352A led to normal recombinant protein expression, and its functions were preserved. Replacing N352 with both S and I led to a loss of N-linked glycan attachment, resulting in a destabilized protein structure with compromised protein function (Figure 5 and Table 1).

Recombinant protein L353P was not expressed in HEK293T cells, which aligns with the prediction that N-glycan can attach in the NXS/T sequon, where X can be any aa except a P. Proline (P) separates the acceptor N from the hydroxyl moiety of S or T in the sequon and inactivates the acceptor sequence (Figure 5F), forming the structural basis for excluding P residues at the middle position (13, 24).

### N-glycosylation site N253

#### Experimental design.

**Figure 6:**
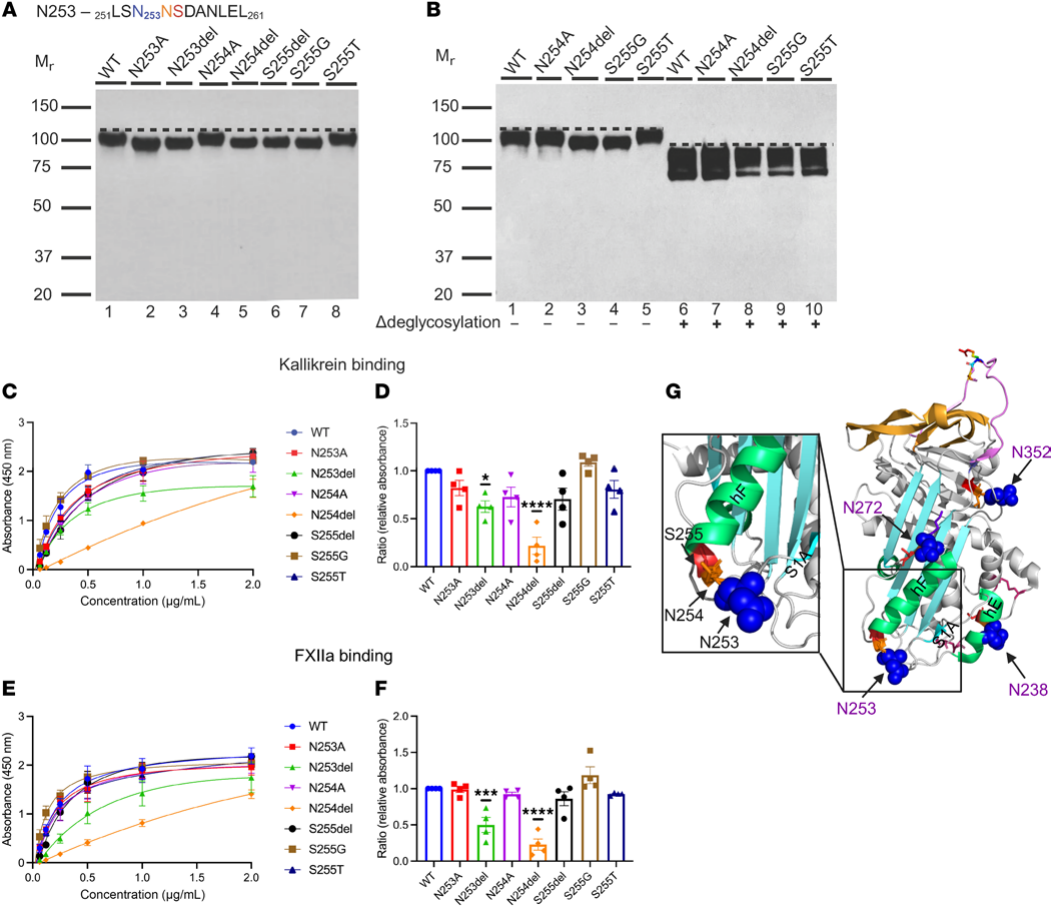
N253 glycosylation site variants. (**A**) WB analysis of supernatants from transfected WT and N253 variant constructs under reducing conditions. Recombinant expression of N253 variants was comparable to WT (see Table 1). N253A, N253del, N254del, and S255G disrupt the N-glycan attachment and lead to a slightly lower M_r_ protein compared with WT. The consensus sequence of N253 glycosylation (NXS/T) is highlighted in blue, orange, and red. It contains 2 Ns in this sequence. (**B**) WB analysis of variants N254del and S255G before and after treatment with glycosidases. Recombinant expression of N254del and S255G was comparable to WT, but with a slightly lower M_r_ compared with WT (lane 1). After deglycosylation, WB demonstrated that N254del (lane 8), S255G (lane 9), and WT have the same M_r_. Δ, post-degylcosylation. (**C**–**F**) Functional analysis of the N253 glycosylation site variants. (**C** and **E**) Absorbance is plotted against protein concentrations. (**D** and **F**) Binding affinity of N253 glycosylation site variants for PKa and FXIIa compared with WT. The binding affinity of N253A, N254A, S255del, S255G, and S255T for PKa and FXIIa was comparable to WT. N253del and N254del exhibited mildly impaired binding to PKa and FXIIa. Results shown are from 3 independent experiments. Data represent mean ± SEM. *P <0.05, ***P < 0.001, ****P < 0.0001 by 1-way ANOVA and Dunnett’s multiple-comparison test. (**G**) Structural analysis of N253. The structure of active C1-INH is shown in a cartoon representation (PDB: 5DU3). N253 is located in the loop connecting strand 1 in β-sheet A (S1A) and helix F (hF).

The consensus sequence at the N253 position is _251_LSN_253_NSDANLEL_261_ (Figure 6). In this sequon, we engineered N253A and N253del at the N253 glycan site and N254A and N254del at the +1 position N254. Additionally, we introduced S255del, S255G, and S255T at the +2 position of the sequon.

#### Genetic analysis.

S255G is reported in gnomAD with an AF of 0.00014% and in silico predictions indicate this change is benign. S255T is reported in LOVD from a patient with HAE who also has a frameshift deletion in exon 7 of the *SERPING1* gene (25). This variant has an AF of 0.0027% in gnomAD and in silico predictions suggest this missense variant is benign (22, 26). The available evidence is currently insufficient to determine the role of S255T in HAE; therefore, it is categorized as a variant of uncertain significance (VUS).

#### Antigenic and functional analyses.

Replacing N253 and N254 with A did not alter recombinant protein expression or secretion (Table 1). Also, deleting N253, N254, and S255 did not affect protein expression (Table 1). Except for variants N254A and S255T, the recombinantly expressed protein from all other variants, including N253A, N253del, N254del, S255del, and S255G, migrated at a slightly lower M_r_ on WB compared with WT, likely due to loss of N-glycan attachment (Figure 6A). After deglycosylation, N254del and S255G ran at the same M_r_ as WT (Figure 6B).

Our functional analysis indicates that N253A and N254A have normal binding affinities for C1s̄, PKa, and FXIIa. N253del and N254del had a decrease in binding to PKa and FXIIa (Figure 6, C–F). However, their binding to C1s̄ was normal (Table 1). The binding affinity of S255del, S255G, and S255T for PKa, FXIIa, and C1s̄ was normal (Figure 6, C–F).

#### Structural analysis.

N253 and N254 are located at the transition from the loop to hF. The replacement of N to A at N253 and N254 results in a change from hydrophilic N to hydrophobic A, which might alter the packing and orientation of the loop. S255 is located in the beginning section of the hF. Glycine (G) is the smallest aa, with only 1 side chain of hydrogen. Due to its size and being at the transition from the loop to hF, the replacement of S255 with G would not interfere with the hF backbone packing (27). In the case of S255T, S and T are both neutral and polar and the change from S to T was structurally tolerable (Table 2) (28).

#### Implications.

The consensus sequence for the N_253_NS glycosylation site displayed different variant expression and functional profiles compared with N238 and N352. The deletion of N at the N238 or N352 sequon completely abolished protein expression. Furthermore, the deletion of S240 and S354 at the N_238_AS and N_352_LS sequon, respectively, prevented expression (Figure 4A and Figure 5A). N253del or N254del in the N_253_NS sequon, however, did not alter the recombinant protein expression, but caused a decrease in both PKa and FXIIa binding (Figure 6 and Table 1). Similarly, S255del led to the loss of the N-glycan site, but did not affect the recombinant variant protein expression or function (Figure 6, A and C–F). These changes are likely due to the secondary structure of the N_253_NS sequon in C1-INH. N253 and N254 are at the loop transitioning into hE, whereas S255 resides in hE. The deletion of N253 or N254 shortens the loop, linking S1A and hE, which leads to a decreased mobility of S1A and hE during RCL insertion. However, the deletion of S255 did not disrupt hE packing (Figure 6G).

In the case of S255T, according to the consensus sequence for N-glycosylation, NXS/T, the replacement of S with T hypothetically should not affect the glycol attachment at the N253 site. Our data reveal that the recombinant protein of S255T exhibited the same M_r_ compared with the WT (Figure 6A), confirming that the replacement of T with S does not affect the N-glycan attachment at the N253 position (26).

### N-glycosylation site N272

#### Experimental design.

**Figure 7:**
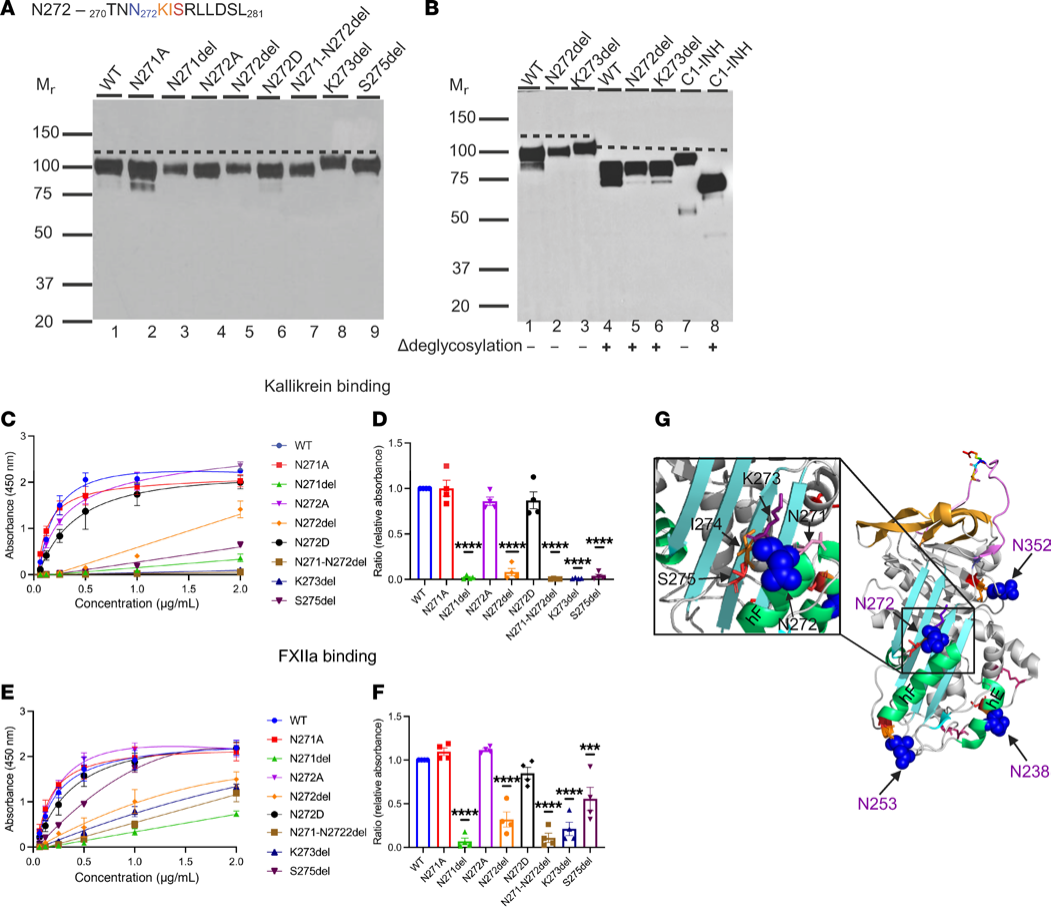
N272 glycosylation site variants. (**A**) WB analysis of supernatants from transfected WT and N272 variant constructs under reducing conditions. Recombinant expression of N271del (lane 3) and N272del (lane 5) was decreased. N271A, N272A, N272D, N271-N272del, K273del, and S275del (lanes 2, 4, 6, 7, 8, and 9) had normal secretion comparable to WT (lane 1). See Table 1. N272-linked glycan site has an atypical N-glycosylation consensus sequence, NNKIS, which is highlighted in blue, purple, orange, and red. (**B**) WB analysis of variants N272del and K273del before and after treatment with glycosidases. Before treatment, K273del (lane 3) had a slightly higher M_r_ compared with N272del (lane 2). After treatment, WB demonstrates that N272del (lane 5) and K273del (lane 6) had the same M_r_. Human purified C1-INH was used as a positive control (lane 7, before treatment; lane 8, after treatment). Adopted from Ren et al. (6). Δ, post-degylcosylation. (**C**–**F**) Functional analysis of the N272 glycosylation site variants. (**C** and **E**) Absorbance is plotted against protein concentration. (**D** and **F**) Comparison of PKa and FXIIa binding between WT and N272 glycosylation site variants. The binding affinity of N271A, N272A, and N272D for PKa and FXIIa was comparable to WT, whereas N271del, N272del, K273del, and N271-N272del exhibited impaired binding activity to both substrates. Interestingly, S275del exhibited a markedly decreased binding to PKa, but not to FXIIa. Data represent mean ± SEM of 3 separate experiments. ***P < 0.001, ****P < 0.0001 by 1-way ANOVA with Dunnett’s multiple-comparison test. (**G**) Structural analysis of N272del and K273del. K273 is located in the loop immediately after hF. The deletion of K273 does affect the conformation of hF. K273del, previously reported, results in a new N-glycosylation site in C1-INH (37). The N271 residue is shown in pink, N272 in blue sphere, and K273 in purple.

The sequence at the N272 site is _270_TNN_272_KISRLLDS_280_ (Figure 7). K273del is reported in patients with HAE and noted to have a higher M_r_ by creating an additional N-glycosylation site at N272 (6). Given that this site contains 2 Ns, we constructed N271A, N271del, N272A, N272del, and S275del. Additionally, we engineered variants N271-N272del and N272D, which are reported by LOVD.

#### Genetic analysis.

N271-N272del, N272del, and K273del are reported in multiple HAE patient studies (5, 29–33). However, these 3 variants are absent in general population databases (gnomAD). N272D is reported in gnomAD with an AF of 0.0003% and in silico analyzes predict that the replacement of N with D is likely benign.

#### Structural analysis.

K273 is in the loop connecting hF and S3A. Deletion of K273 creates an additional N-glycosylation site on N272 by changing the aa sequence NN_272_KIS to an N-glycosylation sequon, NN_272_IS. Adding an N-glycan at the N272 position reduces the flexibility of the hF/S3A loop, which is critical for the RCL insertion during the transition from an active to a latent state (Figure 7G).

#### Antigenic and functional analyses.

The secretion of the recombinantly produced variants N271del and N272del was reduced compared with WT (Table 1). The expression levels of variants N271A, N272A, N272D, N271-N272del, K273del, and S275del were normal (Figure 7A).

The N271del, N272del, and N271-N272del variant proteins exhibited a decrease in binding to C1s̄, FXIIa, and PKa. N271A, N272A, and N272D demonstrated normal binding to their substrates compared with WT (Figure 7, C–F). Deleting S275 did not change the recombinant protein expression; however, the binding of S275del to PKa was markedly reduced, but not to C1s̄ or FXIIa. The mechanism of this selective reduction in binding with PKa is under investigation.

#### Implications.

A mass spectrometry study reported that the SERPIN domain of C1-INH carries 3 N-glycosylation sites, N238, N253, and N352 (14). By deleting K273, a glycosylation sequon is created at the N272 position, NN_272_IS. The variant protein K273del’s binding affinity for C1s̄, PKa, and FXIIa was reduced secondarily to an additional N-linked glycan (Figure 3A and Table 2).

## Discussion

Dysfunctions in C1-INH lead to recurrent and unpredictable episodes of soft tissue swelling in HAE, which can be debilitating and life threatening. While N-glycosylation can play a key role in stabilizing and folding of proteins, little is known about the role of N-glycosylation in the functionally relevant SERPIN domain of C1-INH. Therefore, we sought to better understand the role of N-glycosylation sites in C1-INH protein expression and function. We strategically evaluated variants at or in close proximity to the 3 N-linked glycan sequons in the SERPIN domain. Through this study, we determined that the loss of an N-linked sugar at N238, N253, or N352 is pathologic (Table 1).

The exact molecular determinants that alter N-glycan co- and posttranslational modifications are unclear (34). OST has 2 isoforms, STT3A and STT3B, with different roles in mediating N-linked glycosylation. The STT3A isoform is responsible for the cotranslational modification of the NXS/T sequon in which the nascent polypeptide enters the lumen of the endoplasmic reticulum (ER). STT3B is mainly responsible for posttranslational N-glycosylation, modifying consensus sites that are not glycosylated by STT3A. The depletion of STT3A, but not STT3B, results in the induction of the unfolded protein response pathway. This pathway involves downregulating the transcription of secretory proteins and increasing the removal of misfolded proteins through ER-associated degradation (35–38).

In this study, we first addressed whether the lack of N-glycosylation alters C1-INH protein production. The deletion of an N residue at N_238_AS or N_352_LS resulted in the abolition of recombinant C1-INH protein expression by HEK293T cells. Furthermore, C1-INH variants with an S deletion at the +2 position (at these same loci), including S240del and S354del, were also not expressed. Interestingly, N238A and N352A mutants displayed preserved protein expression and functional levels despite lacking N-glycan attachment.

**Figure 8:**
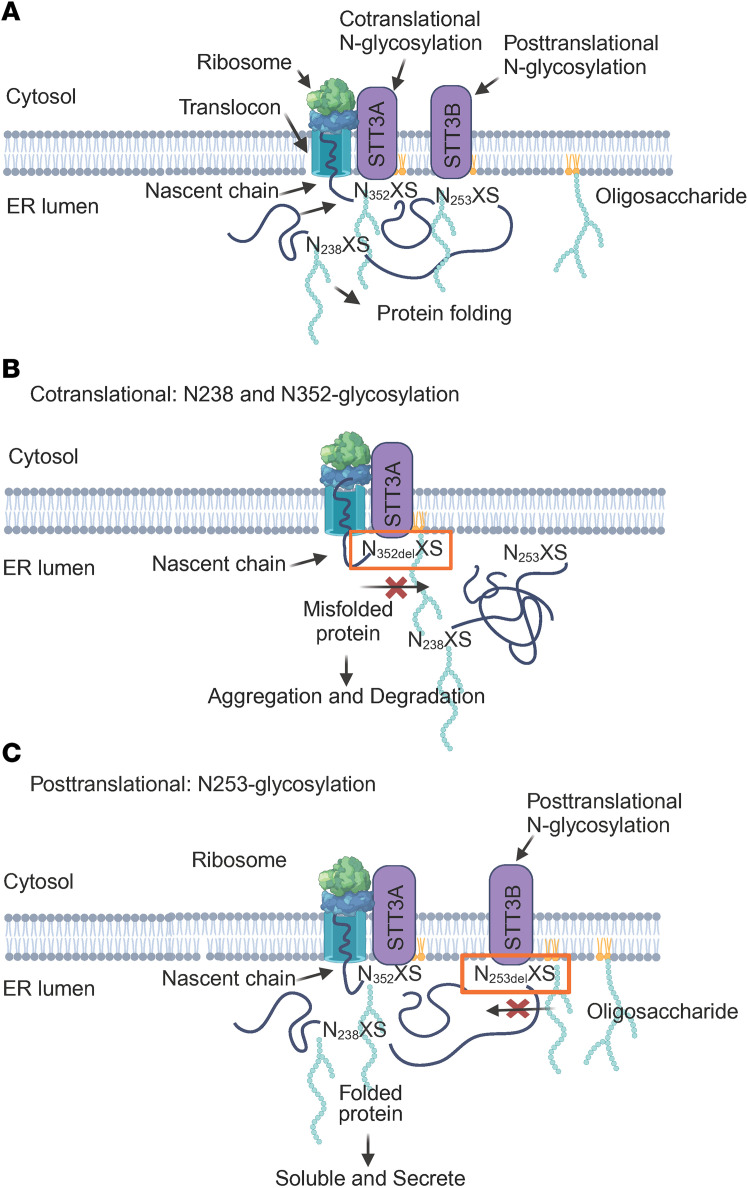
N-glycosylation at N238, N253, and N352 in the ER. (**A**) STT3A cotranslationally transfers an oligosaccharide to N238 and N352 (NXS) in the nascent protein chain. STT3B transfers the oligosaccharide to the N253 sequon in a posttranslational manner. (**B**) Misfolded N352del triggers cotranslational protein degradation (49). (**C**) With preserved protein structure, 253del, even without posttranslational modification, is able to pass the quality control system and be transported out of the ER.

These data suggest that the N-glycosylation sites at N_238_AS and N_352_LS sequons are likely required for cotranslational N-glycan modification. The replacement of N with A at the N_238_ and N_352_ sequons is tolerable structurally and these variants could undergo folding and being exported out of the ER. However, errors in protein folding (misfolded protein) due to N and S deletion likely triggered protein cotranslational degradation (Figure 8) (39, 40).

Unlike the N-glycosylation sites at the N_238_AS and N_352_LS sequons, the site at N_253_NS contains 2 Ns and exhibits different patterns. When N residues were deleted at the N_253_NS site, the variants N253del and N254del showed normal recombinant protein expression comparable to WT. Further S deletion at the +2 position of the N_253_NS sequon also resulted in a preserved protein expression comparable to WT. All 3 mutants migrated at a lower M_r_ on WB than WT due to a lack of N-linked glycan attachment at N253 (Figure 6).

The influences of the middle “X” residue in the N-glycan sequon, NXS, have been studied. If X is a small, non-charged aa, it can be N-glycosylated in an efficient cotranslational manner. In contrast, consensus sites with a bulky hydrophobic, negatively charged middle X residue, in close proximity to an NXS acceptor site or within the cysteine-rich domain, often result in a higher percentage of N-glycans being added via posttranslational modification (26, 41, 42). We hypothesize that the N-glycosylation at N_253_NS occurs during posttranslational modification and the deletion of either N is tolerable without disrupting protein structural folding. Therefore, the recombinant protein was synthesized and secreted despite lacking an N-glycan modification (Figure 6).

NXS/T sequons are highly conserved in the SERPIN domain of C1-INH. At the N_352_XS/T sequon, variant L353P is not expressed in HEK293T cells. This is consistent with the prediction that at the X position in the NXS sequon, it can be any aa, but not P, as it physically prohibits N-linked sugar attachment. Additionally, the consensus NXS sequon indicates that S can be replaced by T or, less often, C. In the case of the N_253_NS sequon, the variant S255T exhibited normal protein expression and function (Figure 6), supporting the conservation of the NXS/T sequon.

N-glycosylation sites N238, N253, and N352 are located on the SERPIN domain of C1-INH and have been confirmed by mass spectrometry (14). The N272 glycosylation site is noted in *The Complement Factsbook* (13) (see Figure 1). However, this N-glycan location site in human C1-INH has not been verified by mass spectrometry. Our understanding of the impact of glycosylation modifications on C1-INH is currently limited. The recombinant C1-INH produced in the mammary gland of transgenic rabbits is available for treating acute lesions in HAE (43, 44). Due to a lower degree of glycosylation, its efficiency suffers from an extremely short half-life of 2.4–2.7 hours, compared with human plasma–derived C1-INH, with a half-life of 56–72 hours (43). A question our study investigated is whether an increased number of glycosylation sites would better facilitate the C1-INH function. This was further “sparked” when we identified 2 unrelated HAE patients carrying heterozygous *SERPING1* variants affecting neighboring N272 and K273. The patient carrying N272del presented as a TYI HAE laboratory phenotype with a low C1-INH serum level, whereas the patient with K273del demonstrated a TYII phenotype with a normal C1-INH serum level, but with dysfunctional C1s̄ binding (6). The deletion of K273 created a new N-glycosylation site as the N_272_IS sequon. The recombinantly expressed K273del protein exhibited an increased M_r_ compared with WT, likely due to another N-linked glycan attachment (Figure 3A and Figure 7, A and B). Further functional analyses of K273del demonstrated impaired binding activity to C1s̄, PKa, and FXIIa. The presence of the additional N-linked glycan likely hindered the insertion of the RCL between S3A and S5A (Figure 2 and Figure 7G). This insight, gained from the additional N-glycan site in K273del, deepened our understanding of the impact of N-glycosylation sequons on C1-INH protein function and may be valuable for future consideration in protein modification and engineering (6, 45) of N-linked glycosylation sites.

The growing application of next-generation sequencing and exome or whole-genome sequencing in investigating rare diseases has led to the identification of an increased number of variants in the *SERPING1* gene (46). To date, more than 1,100 genetic variants have been reported, among which about one-third are SNVs resulting in missense mutations (33, 46). Assessing the pathogenicity of these variants can be challenging, as more than one-half of them are classified as a VUS (46).

In this study, we conducted strategic analyses to examine 11 reported SNVs near the 3 N-glycan sites in the SERPIN domain. Among these, 5 variants, A239D, S255G, N272D, N352S, and S354G, are reported in gnomAD without an accessible clinical history. The other 6 variants, S255T, N352I, L353P, N271-N272del, N272del, and K273del, are reported in patients with HAE (Tables 1 and 2). At the N352 glycosylation site, both N352I and L353P are likely pathogenic (Table 2). N352I exhibited markedly decreased protein expression and L353P was not expressed. At the N253 glycosylation site, 2 variants are likely benign, S255G and S255T, with normal recombinant protein expression and binding affinity for PKa, FXIIa, and C1s̄ (Figure 6C). At the N272 site, K273del has an additional N-glycan site and was dysfunctional, and N272del had low protein expression and function. N271-N272del is a rare variant reported in TYI HAE patients from a Macedonian cohort (30). Recombinant protein expression of N271-N272del was normal, but its binding affinity for C1s̄, PKa, and FXIIa was decreased. N271 and N272 are in the loop connecting hF and S5A, conserved aa in the SERPIN domains (Supplemental Figure 1; supplemental material available online with this article; https://doi.org/10.1172/jci.insight.185548DS1). The deletion of N271-N272 likely disrupts the packing of S5A/hF and further destabilizes the SERPIN domain folding and function (Figure 7G).

It is worth noting that variants S275del and N352S show normal protein expression, but selectively impaired PKa binding. The mechanism of the selectively impaired SERPIN inhibition is not well understood, although it was reported in a study of 12 C1-INH P1 variants (16). The inhibitory activities of 12 R466 variants at the P1 position of the RCL in the SERPIN domain were tested with C1s̄, FXIIa, PKa, and plasmin. Selectively impaired binding activity of P1R466K was observed with FXIIa, less with PKa, and not with C1s̄ or plasmin (16). Currently, the diagnosis of HAE is based on abnormal complement laboratory studies and genetic testing is not routinely performed. The functional analysis of C1-INH is only commercially available to assess its binding to C1s̄, but not to other substrates, such as PKa, FXIIa, and thrombin. Various HAE therapies are available, including C1-INH replacement, PKa inhibitors, FXIIa inhibitors, antifibrinolytics, and B2R antagonists, with a high average cost of $700,000 per patient-year (47). Managing the disease effectively with optimal treatment choices continues to be a persistent challenge. Thus, these findings provide valuable insights for assisting the formulation of personalized treatment by opening up the possibility of selectively choosing a medicine to directly target a specific defect that is impaired in patients with a C1-INH variant.

This investigation focuses on analyzing the impact of N-glycosylation in the SERPIN domain by examining the expression, structure, and function of *SERPING1* variants. The results are most consistent with a pathologic consequence (i.e., HAE) if a missense mutation alters/deletes an N-linked sugar at 4 distinct sites in the SERPIN domain of the C1-INH protein. Our findings suggest that N238 and N352 glycosylation sites undergo cotranslational modification mediated by the STT3A complex, adding oligosaccharides to the nascent protein during its insertion into the ER. This process is crucial for assisting nascent protein folding and transportation (48) (Figure 8A). The failure of attaching N-glycans to these 2 sites can lead to protein misfolding and trigger cotranslational protein degradation (Figure 8B) (49). Conversely, we postulate that the N253 glycosylation site is modified through posttranslational modification mediated by the STT3B complex. N253 is situated between the N_238_AS and N_352_LS sequons that promote skipping by the STT3A complex. The posttranslational N-glycosylation at N253 is required, but not necessary, to acquire the native protein structure. In the absence of N253-glycan attachment, the effect is tolerable compared with the N238 and N352 sites (Figure 8C) (37, 49, 50). N253 site variants have preserved expression levels comparable to WT (Figure 6). In addition, our finding demonstrates that adding an additional N-glycosylation site can be deleterious by interrupting the SERPIN domain function. The scope of this study is limited to only analyzing the N-glycosylation sites in the SERPIN domain. Further research is warranted to understand the structure and function of the N-glycosylation sites in the NTD.

## Methods

### Sex as a biological variable

As only HEK293T cells were used in this study, sex was not considered as a biological variable.

### Preparation and expression of variants

The *SERPING1* pcDNA3.1 expression vector (Genescript) was used to create the C1-INH variants. The variants were produced using the QuikChange XL site-directed mutagenesis kit (Agilent Technologies). Each *SERPING1* cDNA clone was sequenced. The variants were transiently transfected into HEK293T cells (ATCC, CRL-3216) using the Xfect reagent (Takara Bio USA) where Dulbecco’s modified Eagle medium (DMEM) was replaced with OptiMEM (Invitrogen). Each transfection experiment was conducted in 3 independent biological replicates. Supernatants were collected after 48 hours, concentrated 40-fold, and then stored in aliquots at –80°C (51).

### Quantification and WB

The quantity of each recombinant C1-INH variant protein was determined by ELISA according to the manufacturer’s recommendations (Abcam). Electrophoretic patterns were evaluated and compared to WT using transfectant supernatants that were analyzed under reducing conditions using 4%–20% SDS-PAGE, transferred to nitrocellulose membranes, and then probed with 1:1,000 rabbit anti–human C1-INH mAb (Abcam, AB134918) as the primary Ab, specifically recognizing the NTD of C1-INH between amino acids 22 and 100, followed by a 1:10,000 horseradish peroxidase–conjugated (HRP-conjugated) goat anti–rabbit IgG (Abcam, AB205718).

### C1s̄, FXIIa, and PKa binding assays

#### C1s̄.

The binding of C1s̄ to C1-INH was measured according to the manufacturer’s instructions (Quidel). In the first step, standards, controls, and C1-INH variants were incubated with biotinylated C1s̄. Next, the incubation mixtures containing the C1s̄/C1-INH complex were added to microtiter wells precoated with streptavidin. After incubation, the wells were washed 3 times to remove unbound protein. Then, the goat anti–human C1-INH Ab was added to each test well to bind with the C1s̄/C1-INH complex captured on the surface of the streptavidin-coated microtiter wells. After washing, the HRP-conjugated goat anti-human Ab was added to each microassay well. After adding 3,3′,5,5′-tetramethylbenzidine dihydrochloride (TMB) substrate, the complex generated a yellow color, the intensity of which was measured spectrophotometrically at 450 nm. The relative absorbance was calculated as the absorbance for each C1-INH variant divided by the absorbance of WT at protein concentrations of 125 ng/mL, 250 ng/mL, 500 ng/mL, and 1 μg/mL.

#### FXIIa and PKa.

Purified human FXIIa and PKa were obtained from Enzyme Research Laboratories. The biotinylation kit and streptavidin-coated plate were obtained from Thermo Fisher Scientific. FXIIa and PKa were biotinylated according to the manufacturer’s recommendations. In brief, FXIIa and PKa were dissolved in phosphate-buffered saline (PBS) and then incubated with sulfo-NHS-LC-biotin on ice for 2 hours. Excess nonreacted and hydrolyzed biotin was removed through a spin column and the biotin-labeled protein concentrations were measured by NanoDrop. ELISA was utilized to assess the binding of WT and variant C1-INH to FXIIa and PKa, as described previously (20). Biotinylated FXIIa and PKa (25 μL of 2 μg/mL), an equivalent molar amount of C1-INH, and 50 μL of reaction buffer (2% BSA in PBS with Tween [PBS-T]) were added to the streptavidin-coated plate and incubated for 1 hour at 37°C. The bound PKa-C1-INH or FXIIa-C1-INH complex was detected by 1:10,000 mouse anti–C1-INH mAb (Abcam, ab244029), followed by incubation at room temperature for 1 hour. After incubation, the plates were washed 3 times using PBS-T (300 μL/each). A 1:10,000 dilution of HRP-conjugated goat anti–rabbit IgG (Abcam) secondary Ab was added and then incubated at 37°C for 1 hour. The detection of bound C1-INH complex was carried out as described previously (20). On at least 3 occasions, binding assays were performed with serially diluted samples. C1s̄, FXIIa, and PKa binding assays were repeated a minimum of 3 times for each variant.

#### Molecular modeling.

PyMOL v3.0 (https://www.pymol.org) was employed to visualize and analyze the protein structures.

#### Data availability.

Raw data for all graphs are reported in the Supporting Data Values file. All gel data and WBs in this study are presented in the full, unedited gel file available in the Supplemental Data with full annotations.

#### Statistics.

Statistical analyses were performed using Prism 10 (GraphPad). Comparisons between 2 groups were assessed using a paired *t* test (nonparametric). Comparisons among groups were performed using a 1-way ANOVA with Dunnett’s multiple-comparison test. A *P* value of less than 0.05 was considered significant.

## Author contributions

ZR and JPA conceived and designed the experiments, analyzed the data, and interpreted results. ZR and JB performed the experiments. ZR conducted the structural analyses. SZ classified variants in accordance with American College of Medical Genetics 2015 criteria. ZR prepared the figures and drafted the manuscript. ZR, JB, SZ, NP, HJW, and JPA edited the manuscript.

## Supplementary Material

Supplemental data

Unedited blot and gel images

Supporting data values

## Figures and Tables

**Table 1:**
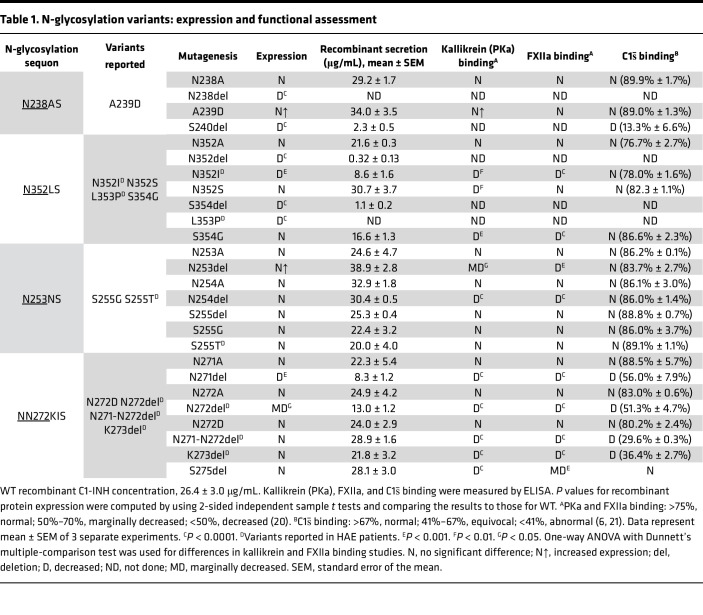
N-glycosylation variants: expression and functional assessment

**Table 2:**
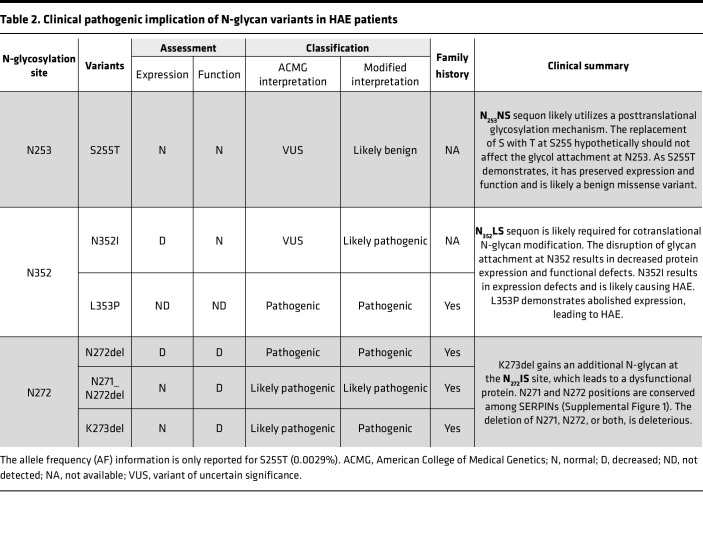
Clinical pathogenic implication of N-glycan variants in HAE patients

## Version 1 01/17/2025

In-Press Preview

## Version 2 02/24/2025

Electronic publication
