# Supplementary material for: N-glycosylation in the SERPIN domain of the C1-esterase inhibitor in hereditary angioedema
Source: JCI Insight. 2025 Jan 16;10(4):e185548. doi: 10.1172/jci.insight.185548 (PMC11949052; doi:10.1172/jci.insight.185548)
Supplement: Supplemental data [file jciinsight-10-185548-s262.pdf]

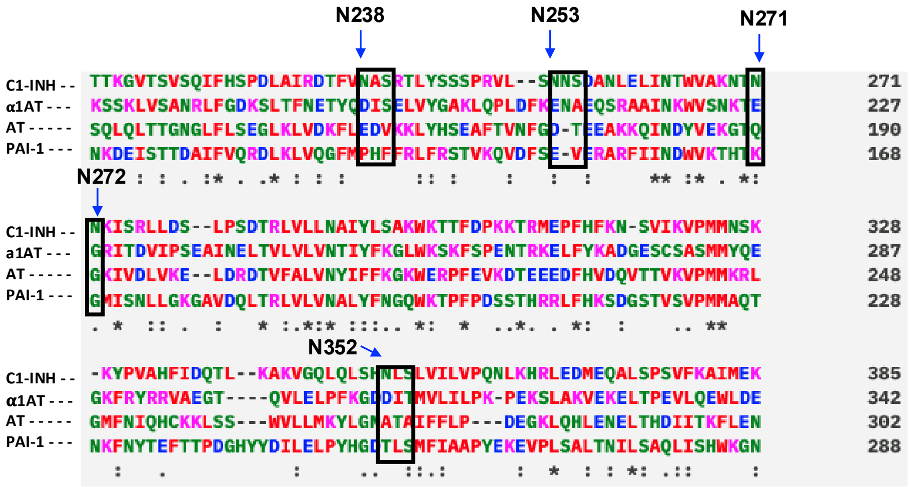

Supplemental Figure 1. Amino acid sequence alignment of selected SERPIN proteins. Sequence alignments were generated with NCBI protein blast software Clustal Omega. Red color, hydrophobic; blue, acidic; magenta, basic; and green, hydroxyl, sulfhydryl, or amine residues. C1-INH, C1 esterase inhibitor; α1AT, α1-antitrypsin; AT, antithrombin; PAI-1, plasminogen activator inhibitor 1. "*" positions with a single, fully conserved residue. ":" positions with conserved amino acids bearing similar properties. "." positions with conservation between amino acid groups with weakly similar properties.
