# Supplementary material for: N-glycosylation in the SERPIN domain of the C1-esterase inhibitor in hereditary angioedema
Source: JCI Insight. 2025 Jan 16;10(4):e185548. doi: 10.1172/jci.insight.185548 (PMC11949052; doi:10.1172/jci.insight.185548)
Supplement: Unedited blot and gel images [file jciinsight-10-185548-s263.pdf]

Full unedited gel for Figure 4A, lanes used (1-5)

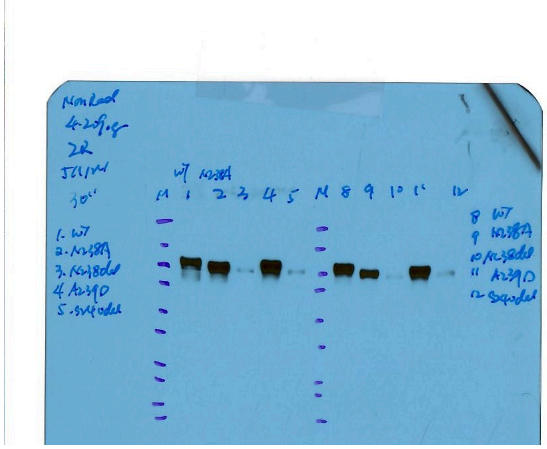

4-20% SDS-PAGE

Primary Ab: 1:1,000 rabbit anti-human C1-INH mAb (Abcam AB134918), secondary: 1:10,000 horseradish peroxidase (HRP)-conjugated goat anti-rabbit IgG (Abcam).

Full unedited gel for Figure 5A, lanes used (1-8)

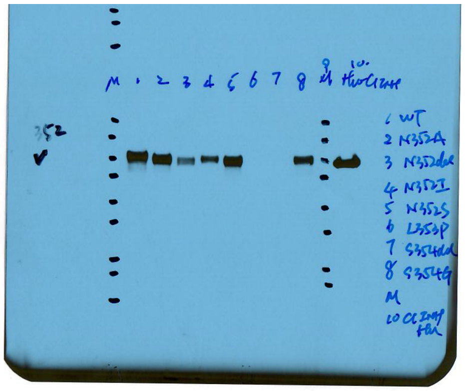

4-20% SDS-PAGE

Primary Ab: 1:1,000 rabbit anti-human C1-INH mAb (Abcam AB134918), secondary: 1:10,000 horseradish peroxidase (HRP)-conjugated goat anti-rabbit IgG (Abcam).

Full unedited gel for Figure 6A, lanes used (1-8)

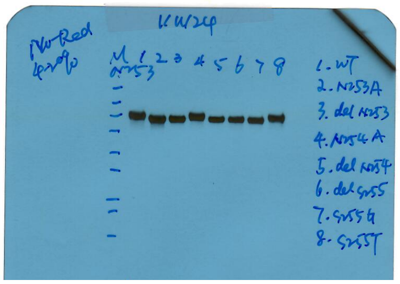

4-20% SDS-PAGE

Primary Ab: 1:1,000 rabbit anti-human C1-INH mAb (Abcam AB134918), secondary: 1:10,000 horseradish peroxidase (HRP)-conjugated goat anti-rabbit IgG (Abcam).

Full unedited gel for Figure 6B, lanes used (1-10)

Pre-deglycosylation

1. WT
2. N254A
3. N254del
4. S255G
5. S255T

Post-deglycosylation
WT

1. WT
2. N254A
3. N254del
4. S255G
5. S255T

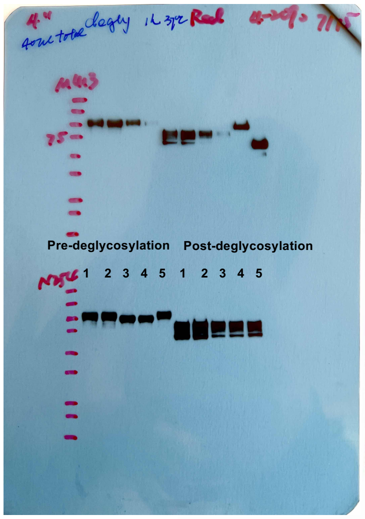

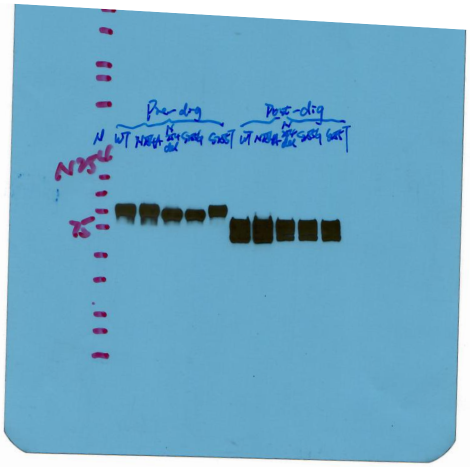

4-20% SDS-PAGE

Primary Ab: 1:1,000 rabbit anti-human C1-INH mAb (Abcam AB134918), secondary: 1:10,000 horseradish peroxidase (HRP)-conjugated goat anti-rabbit IgG (Abcam).

Full unedited gel for Figure 7A, lanes used (1-9)

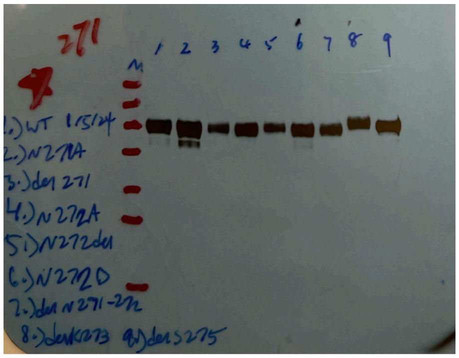

4-20% SDS-PAGE

Primary Ab: 1:1,000 rabbit anti-human C1-INH mAb (Abcam AB134918), secondary: 1:10,000 horseradish peroxidase (HRP)-conjugated goat anti-rabbit IgG (Abcam).

Full unedited gel for Figure 7B, lanes used (1-8)

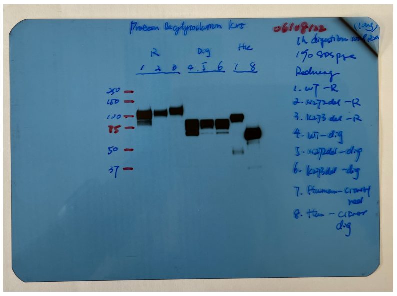

4-20% SDS-PAGE

Primary Ab: 1:1,000 rabbit anti-human C1-INH mAb (Abcam AB134918), secondary: 1:10,000 horseradish peroxidase (HRP)-conjugated goat anti-rabbit IgG (Abcam).
